# What Is the Long-Term Clinical Efficacy of the Thoraflex™ Hybrid Prosthesis for Aortic Arch Repair?

**DOI:** 10.3389/fcvm.2022.842165

**Published:** 2022-02-22

**Authors:** Sven Z. C. P. Tan, Matti Jubouri, Idhrees Mohammed, Mohamad Bashir

**Affiliations:** ^1^Barts and The London School of Medicine and Dentistry, Queen Mary University of London, London, United Kingdom; ^2^Hull York Medical School, University of York, York, United Kingdom; ^3^Cardiovascular Department, SRM Institute of Medical Science, Institute of Cardiac & Aortic Disorders, SIMS Hospital, Chennai, India; ^4^Vascular and Endovascular Surgery, Velindre University NHS Trust, Health Education and Improvement Wales, Cardiff, United Kingdom

**Keywords:** frozen elephant trunk (FET), Thoraflex™, aortic arch, dissection (TAAD), aneurysm

## Abstract

**Background:**

The widespread adoption of the frozen elephant trunk (FET) technique for total arch reconstruction (TAR) in aortic arch aneurysm and dissection has led to the development of numerous commercial single-piece FET devices, each with its own unique design features. One such device, Thoraflex™ Hybrid (Terumo Aortic, Glasgow, Scotland), has enjoyed widespread use since its introduction. We present and appraisal of its long-term clinical efficacy, based on international data.

**Materials and Methods:**

Pre-, intra-, and postoperative data associated with Thoraflex™ Hybrid implantations for aortic arch dissection, aneurysm, and penetrating atherosclerotic ulcer (PAU) up to April 2019 was gathered and is presented herein. Follow-up data at discharge, 3-, 6-, 12-, 24-, 36-, 48-, 60-, 72-, and 84- months post-implantation are included.

**Results:**

Data associated with 931 cases of Thoraflex™ Hybrid implantation are included. Mean age at implantation was 63 ± 12 years. 55% of patients included were male. Aortic dissection accounted for 48% (*n* = 464) of cases. Mean cardiopulmonary bypass and circulatory arrest durations were 202 +72 and 69 ± 50 min, respectively. 30-day mortality was 0.6% (*n* = 6), while overall mortality was 14 (1.5%). Freedom from adverse events at 84 months was 95% (*n* = 869). Postoperative complications included neurological deficit, multi-organ failure, cardiorespiratory compromise, and infection.

**Discussion:**

Thoraflex™ Hybrid's unique design is advantageous in comparison to market alternatives. Our data is consistent with that reported in literature and suggests Thoraflex™ Hybrid is associated with favourable rates of mortality and morbidity.

**Conclusion:**

Thoraflex™ Hybrid remains a central player in the aortic arch prosthesis market. Its use it widespread and is associated with favourable design features and clinical outcomes relative to market alternatives.

## Introduction

The surgical management of aortic arch and thoracic aortic diseases is invariably complex, and often associated with high rates of mortality and morbidity. The frozen elephant trunk (FET) technique evolved from the conventional elephant trunk (cET) technique pioneered by Borst et al. and allows for the single-stage repair of the aortic arch and proximal descending thoracic aorta (DTA) (1). The FET technique has enjoyed widespread adoption in part due to its benefits and surgical straightforwardness relative to the cET technique (2). Total arch reconstruction (TAR) with FET has been reported to attenuate the risk of proximal endoleak and stent migration while promoting distal aortic remodelling (3). The stented (or “frozen”) distal graft introduced anterograde into the distal arch or proximal DTA, combined with aortic arch revascularisation using a surgical polyester graft, offers a single-stage hybrid approach, eliminating both the need for a second procedure and associated interstage mortality (4). Commercially available FET devices provide a single-piece hybrid prosthesis for total arch reconstruction (TAR), negating the need for off-label implantation of commercial thoracic endovascular aortic repair (TEVAR) grafts antegrade through the aortic arch—a method described as being suboptimal and technically awkward, as well as being associated with stent migration, unstable proximal fixation, and type 1A endoleak (4).

The Thoraflex™ Hybrid prosthesis (Terumo Aortic, Glasgow, Scotland) has enjoyed particularly widespread international use, even prior to gaining FDA breakthrough device approval in April 2020 (5). Its innovative and intuitive design has propelled it to the forefront of the aortic arch device market, alongside other established arch devices such as the E-Vita™ family (CryoLife Inc., Kennesaw, GA, USA), Cronus™ (MicroPort Medical, Shanghai, China), and Frozenix™ (Japan Lifeline, Tokyo, Japan). Recent evidence suggests that Thoraflex Hybrid™ is particularly effective in acute type A aortic dissection (ATAAD), and may be associated with lower rates of postoperative complications than E-Vita™ Open (6).

The emergence of and competition between various prominent aortic arch devices necessitates an evidence-based appreciation of each device's benefits, drawbacks, and associated risks. We seek to review and analyse available evidence to evaluate the overall performance of Thoraflex Hybrid™ on the international level, with a view of facilitating informed clinical decision-making on device choice for TAR with FET. The international long-term clinical efficacy of Thoraflex Hybrid™ is evaluated in terms of design features, pre- intra- and postoperative data, and an evaluation of clinical outcomes, namely: mortality, aortic remodelling, neurological outcomes, coagulopathic complications, end-organ compromise, and reintervention rate. The analysis of outcomes associated with Thoraflex Hybrid™ is contextualised and evaluated against those associated with other aortic arch devices.

## Materials and Methods

Baseline characteristics, intraoperative metrics, and postoperative follow-up data were gathered from various aortic centres internationally and are collated below. An appraisal of these data provides an appreciation of the international performance of Thoraflex Hybrid™. Our analysis of clinical outcomes is augmented and contextualised in subsequent sections.

## Results

### Baseline Characteristics

Nine hundred thirty-one patients were implanted with Thoraflex™ Hybrid as of April 2019. The baseline demographics, indications for implantation, and relevant medical history of these 931 individuals are summarised in Table 1. Mean age at the time of prosthesis implantation was 63 ± 12 years. 55% (*n* = 512) of patients treated were male. Male and female patients were on average 59 ± 13 years and 67 ± 9 years old, respectively. Aortic dissection was the predominant indication for aortic repair (*n* = 464, 48%), of which 158 patients presented with ATAAD (35%) and 79 with acute TBAD (18%). 419 (45%) patients presented with aortic aneurysm, while 48 (5%) presented with penetrating atherosclerotic ulcer (PAU). 41 (4%) patients were known to be comorbid with Marfan syndrome, with an average age of 41 ± 13 years, and the youngest was 23 years old. Patients were predominantly classified as ASA grade III or IV (*n* = 503, 54%; and *n* = 205, 22%, respectively).

**Table 1:** Summarised baseline demographic characteristics, indications for use of Thoraflex™, and relevant past medical history.

**Baseline characteristics, indications, medical history**			
	**Variable**		**Value**
*n* = 931	Age (years)		6 ± 12
	Male		6 (55%)
	Female		6 (45%)
	Height (cm)		6 ± 11
	Weight (kg)		6 ± 15
	ASA grade	I	28 (3%)
		II	130 (14%)
		III	503 (54%)
		IV	205 (22%)
		V	65 (7%)
	**Indication**		**Value**
*n* = 931	Aneurysm		419 (45%)
	PAU		48 (5%)
	Dissection		464 (50%)
		Acute Type A	158 (35%)
		Acute Type B	79 (18%)
		Acute (unspecified)	28 (6%)
		Chronic Type A	112 (24%)
		Chronic Type B	70 (15%)
		Chronic (unspecified)	19 (4%)
	**Pre-existing condition**		**Value**
*n* = 376	Previous surgery		6 (19%)
	Aortic valve insufficiency		6 (11%)
	Marfan syndrome		6 (4%)
	Myocardial infarction		6 (2%)
	Cerebral aneurysms		6 (0.5%)
	CVA		6 (0.5%)
	Paresis/Paraplegia		6 (2%)
	TIA		6 (1%)

### Intraoperative Data

Intraoperative data on all 931 patients are summarised in Table 2. Average CPB, aortic cross-clamp, circulatory arrest, and ACP times were 202 ±7 2, 145 ± 63, 69 ± 50, and 90 ± 44 min, respectively. Thoraflex™ deployment time was on average 3 ± 3 min. The majority of patients (*n* = 512 [55%]) underwent concomitant procedures, including aortic valve resuspension, coronary artery bypass grafting, and Bentall procedure.

**Table 2:** Summary of intraoperative data of all 931 patients implanted with Thoraflex™.

**Intraoperative data**		
**Variable**		**Value**
*n* = 931	CPB time (min)	202 ± 72
	AXC time (min)	145 ± 63
	HCA time (min)	69 ± 50
	ACP time (min)	90 ± 44
	Time to deploy Thoraflex™ Hybrid graft (min)	3 ± 3
	Total duration of operation (min)	329 ± 112
	Lowest core temperature (°C)	24 ± 3
	Concomitant surgery (n)	512 (55%)

### Follow-Up Data

Follow-up data from each centre were obtained. Follow-up points were set at discharge, 3-, 6-, 12-, 24-, 36-, 48-, 60-, 72-, and 84- months post-implantation. Tables 3–5 summarise post-implantation mortality and complication rates. Overall, 30-day mortality was 0.8% 7 (*n* = 7), while freedom from adverse events at discharge and at 3, 6, and 12 months was 96% (*n* = 891), 96% (*n* = 890), and 95% (*n* = 887), respectively. 21% (*n* = 3) of all 14 postoperative mortalities across the 84-month follow-up duration were attributed specifically to the device used for aortic repair (Thoraflex Hybrid™), while 79% (*n* = 11) other mortalities across the 84-month follow-up period were procedure-related nor not device-related. Freedom from adverse events remained at 94% (*n* = 869) up to 84 months post-implantation. Analysis of post-implantation complications and overall mortality rates facilitates and evaluation of the international performance of Thoraflex Hybrid™; these are discussed in subsequent sections.

**Table 3:** Summary of event-free survival rates at pre-set follow-up points.

**Freedom from adverse events**										
**Follow-up point (Months)**	**Discharge** **(***n =*** 931)**	**3 (***n =*** 925)**	**6 (***n =*** 925)**	**12 (***n =*** 924)**	**24 (***n =*** 924)**	**36 (***n =*** 924)**	**48 (***n =*** 924)**	**60 (***n =*** 924)**	**72 (***n =*** 924)**	**84 (***n =*** 924)**
**Event-free survival** **n (%)**	894 (96%)	891 (96%)	890 (95%)	887 (95%)	886 (95%)	882 (95%)	873 (94%)	869 (94%)	869 (94%)	869 (94%)

**Table 4:** Summary of 30-day mortality causes.

**30-day mortality causes**		
**Postoperative day**	**Cause of death**	**Nature**
0	Multi-organ failure	Procedure related
1	Bleeding, biventricular heart failure	Procedure related
1	Left ventricular dysfunction	Not device related
14	Multi-organ failure	Procedure related
15	Acute Respiratory Distress Syndrome (ARDS)	Procedure related
24	Haemodynamic shock	Procedure related
223	Respiratory failure	Device related
284	Multi-organ failure	Procedure related
316	Bleeding, biventricular heart failure	Procedure related
563	Bleeding, biventricular heart failure	Procedure related
744	Multi-organ failure	Procedure related
812	Respiratory failure	Device related
882	Respiratory failure	Device related
952	Multi-organ failure	Procedure related

**Table 5:** Summary of all postoperative adverse events (*n* = 59).

**Summary of postoperative adverse events**		
**Post-operative day**	**Adverse event**	**Device related?**
**0–30 days postoperative**		
0	Respiratory insufficiency	Not device related
0	Percardial effusion	Not device related
0	Bleeding, immediate rethoracotomy, death	Procedure related
0	Paraplegia	Procedure related
0	Bleeding, pericardial tamponade	Procedure related
0	Drainage of haematoma	Procedure related
0	Haematoma	Procedure related
0	Hoarseness	Procedure related
0	Acute on chronic renal failure	Not device related
0	Acute renal failure	Not device related
0	Acute renal failure	Not device related
0	Bleeding	Not device related
1	Multi-organ failure; sepsis; rhabdomyolysis; death	Procedure related
1	Paraplegia	Not device related
1	Paraparesis	Not device related
1	Pneumothorax	Procedure related
1	Bacterial pneumonia	Not device related
1	Left ventricular dysfunction	Not device related
1	Respiratory insufficiency	Not device related
1	Right ventricular failure	Procedure related
1	Radialis nerve paralysis	Procedure related
1	Infection	Not device related
2	Pneumonia	Not device related
2	Infection	Procedure related
2	Bilateral vocal cord paresis	Procedure related
2	Psychiatric distoriation	Not device related
6	Renal failure; Liver failure; haemodynamic instability and shock	Procedure related
7	Recurrent TIAs	Procedure related
7	Occlusion of left subclavian artery with subclavian steal syndrome	Procedure related
8	Infection	Procedure related
9	Atrial Fibrillation	Not Device Related
9	Suspicion of microembolism in lower arm	Procedure related
11	Fever, infection	Procedure related
11	Sternal instability	Not device related
12	Sepsis, Mediastinitis, Pleural empyema	Procedure related
22	Multi-organ failure	Not device related
26	Thoracic and epigastric pain	Not device related
**30 days–3 months postoperative**		
36	Haemothorax (right side)	Procedure related
41	Hospitalisation because of sternal pain	Not device related
43	Weakness of the left arm	Procedure related
**3–6 months postoperative**		
150	Endoleak (persistent type Ib)	Not device related
**6–12 months postoperative**		
248	Hospitalisation because of sternal pain	Not device related
283	Haematothorax (right side)	Procedure related
341	Weakness of the left arm	Procedure related
**12–24 months postoperative**		
398	Stroke	Procedure related
**24–36 months postoperative**		
514	Acute on chronic renal failure	Procedure related
889	Hematoma	Procedure related
**36–48 months postoperative**		
1,118	Paraplegia	Not device related
1,298	Stroke	Not device related
1,319	Recurrent TIAs	Not device related
1,338	Bacterial pneumonia	Not device related
**48–60 months postoperative**		
1,512	Acute on chronic renal failure	Not device related
1,583	Stroke	Procedure related
1,622	Pneumothorax	Procedure related
1,662	Hematoma	Not device related
1,669	Stroke	Not device related
1,703	Paraplegia	Not device related
1,710	Paraplegia	Not device related
1,771	Recurrent TIAs	Not device related
1,801	Pericardial Effusion	Not device related
**60–84 months postoperative**		
1,918	Infection	Procedure related
2,024	Stroke	Not device related
2,421	Fever, infection	Procedure related
2,501	Acute renal failure	Not device related

## Discussion

### Thoraflex™ Hybrid Design Features

The unique design features of Thoraflex Hybrid™ undoubtedly play a central role in influencing its usability and efficacy in aortic stabilisation and long-term positive remodelling. Several key design features of the device set it apart from market alternatives and improve the ease and safety of its deployment in straightforward but also in challenging anatomies.

Conventional TEVAR grafts were not designed for anterograde introduction into the DTA for TAR—hence such an off-label approach is associated with unstable fixation, migration, and endoleak (4). The proximal sewn graft of Thoraflex Hybrid™, combined with the distal anastomosis cuff, eliminates the risk of endoleak, and stent migration. Stable fixation is augmented by the unique nitinol ringed stent—its geometric configuration allows the stent to conform along the curvature of the native arch and proximal DTA, through most anatomical angulations (4).

The plexus of 4 arch branches originating from the market-leading Gelweave woven polyester graft represent a key advantage over devices such as the E-Vita™ family and Cronus™ (7). Three of the four branches facilitate supra-aortic vessel reimplantation during TAR without the need for the island technique. This is especially advantageous in patients with Marfan syndrome, atherosclerotic aortic arch, and in those with a greater distance between the origins of each arch vessel (8). The adjustable length of each arch branch may also simplify challenging LSA anastomoses, and indeed the availability of a fourth branch allows reconfiguration of arch implantation to improve LSA reimplantation (7, 8). Chu et al. notes that preoperative left carotid-LSA bypass or transposition remains an option in especially challenging cases (4).

Furthermore, the inclusion of the fourth arch branch (a feature unique to Thoraflex Hybrid™) allows lower body perfusion to be restored immediately after distal anastomosis. This may greatly reduce the duration for which the viscera, spinal cord, and lower limbs are exposed to circulatory arrest, and thereby mitigate the risk of ischaemic complications (8, 9).

Thoraflex Hybrid™ is available in a wide range of diameters—the proximal graft and distal stented portions may be configured with different diameters with either a 100 or 150 mm stent length (10). This facilitates a greater level of anatomic similarity with the native aorta, and arguably represents the closest option to readily available custom-made FET grafts. Finally, the Thoraflex Hybrid™ graft is impregnated with gelatine to ensure blood-tightness, preventing catastrophic post-implantation graft oozing, or leakage (11).

### Mortality

TAR with FET, especially in the acute setting, remains associated with high mortality and morbidity rates (12). This is unsurprising considering the haemodynamic significance and anatomical positioning of the aortic arch and DTA, as well as the surgical and anaesthetic complexity of TAR. As of January 2020, over 30,000 hybrid arch FET prostheses have been implanted, and early mortality has ranged from 1.8 to 17.2% across various commercial and non-commercial device configurations (13). International data on the performance of Thoraflex Hybrid™ in terms of mortality are encouragingly positive. As noted in Tables 3, 4, our series featured a 1.5% (*n* = 14) 30-day mortality rate and a 7-year survival rate of 99% (*n* = 924). It is worth noting that only 3 of all deaths highlighted herein have been attributed to the implanted device; all other deaths and adverse events were procedure-related but not- device related. The causes of mortality following TAR with FET are multi-factorial, and include neurological injury, disease progression, end-organ damage, and intraoperative haemorrhage. This is unsurprising given the invasive and radical nature of TAR, as well as the need for CPB, HCA, and adjunctive cerebral perfusion. The 3 device-related mortalities included herein resulted from postoperative respiratory failure, one of which was secondary to spinal cord injury.

Chu et al., in their retrospective Canadian multi-centre study, reported an overall 30-day and in-hospital mortality rate of 5% for TAR with Thoraflex Hybrid™. 30-day, 12-month, and 24-month survival rates were 95, 95, and 90%, respectively. Their cohort spanned 9 different centres, and included acute dissection, acute rupture, chronic dissection, and aneurysm of the aortic arch and DTA. 30% of cases included were emergent salvage procedures, and all cases were conducted under moderate HCA with ACP (4). More recently, Soknes et al. note that from December 2014—December 2019, 34 patients were treated at their Oslo centre with Thoraflex Hybrid™, with an 8.8% 30-day mortality rate. There were 4 further mortalities during follow up (on average, 32.4 ± 19.4 months). One-year survival was 88%, while 3-year survival was 75%. Notably, all early mortalities in this cohort were attributable to stroke or spinal cord ischaemia (SCI) (14). Further, Shrestha et al. (7) report on the first 100 patients treated with Thoraflex Hybrid™ at their Bologna centre reported a 7% 30-day and in-hospital mortality rate, with acute dissection (*n* = 37) being the predominant indication (chronic dissection: *n* = 31; aortic aneurysm: *n* = 31) (7). Interestingly, Di Bartolomeo et al. reported a 0% mortality rate associated with TAR using Thoraflex Hybrid™ at their Bologna centre, though out of their 10-patient cohort, none were acute cases (15). Reports from various international centres on the performance of Thoraflex Hybrid™ reveal varied results yet are consistent with the present series in highlighting that FET with Thoraflex Hybrid™ is associated with relatively favourable mortality and event-free survival rates, especially over a longer-term tenure across complex patient anatomy treatment.

How does Thoraflex Hybrid™ perform against market competitors? In their multi-centre report on TAR with FET, Berger et al. compared outcomes associated with implantation of Thoraflex Hybrid™ (55 patients, Bad Krozingen, Germany and Salzburg, Austria) and E-Vita™ Open (33 patients, Vienna, Austria) for acute aortic dissection. Their findings demonstrated that Thoraflex Hybrid™ was associated with an 11% in-hospital mortality rate, compared to 12% in E-Vita™ Open (16). Ma et al. note that the in-hospital mortality rates associated with E-Vita™ when implanted for acute dissection, chronic dissection, and aortic aneurysm were 18, 17, and 12%, respectively. In contrast, Thoraflex Hybrid™ was associated with an early mortality rate of 6% at one Hannover centre. Notably, Song et al. reported 0 in-hospital mortalities in 16 patients treated with Cronus™ for ATAAD in Xiamen, China, between February 2018 and August 2019; while Charchyan et al. more recently reported that though the difference in mortality rates between Thoraflex Hybrid™ and E-Vita™ for non-acute DeBakey type I aortic dissection were insignificant, Thoraflex Hybrid™ was associated with improved morbidity rates and freedom from dSINE (17, 18).

Having contextualised the cohort of patients in this international appraisal of performance standards of Thoraflex™ Hybrid for arch repair, it remains clear that the prosthesis is associated with significantly lower rates of mortality relative to market counterparts.

### Aortic Remodelling

Aortic remodelling is a key metric by which the success or failure of aortic repair can be gauged: positive remodelling refers to post-FET implantation thrombosis and elimination of the FL, whereas negative remodelling indicates FL or aneurysmal expansion. Remodelling of the aorta post-intervention (open, endovascular, or hybrid) is often quantified in terms of the TL to total aortic diameter ratio, TL to FL diameter ratio, or the presence of FL thrombosis at the level of the stent graft or distal thereto. Aortic remodelling has also been identified as a predictor of yearly aortic growth rates; Fattouch et al. emphasise that patients with persisting FL patency exhibit greater rates of aortic growth than those with FL thrombosis (2.8 ± 0.4 mm vs. 1.1 ± 0.2 mm, respectively) (19). This effect is thought to be especially pronounced around the proximal DTA due to the greater haemodynamic shear stress exerted thereon as a result of its convexity. Indeed, Sakaguchi et al. have identified an aortic diameter >35 mm as being a risk factor for persistent FL patency (20). Follow-up imaging to determine the extent of postoperative aortic remodelling was unavailable in the present series.

However, Shrestha et al. Hannover group reported a significant increase in both the TL diameter and stable aortic diameter around the Thoraflex Hybrid™ stent graft in all patients with acute aortic dissection included in their study. This effect was noted to have remained stable throughout follow up, and a 100% rate of positive aortic remodelling was achieved within 24 months. TL diameter between the distal end of the Thoraflex Hybrid™ stent and the diaphragm was also improved. Similar results were seen in patients with chronic aortic dissection treated with Thoraflex Hybrid™, wherein a decrease in total aortic diameter—driven by FL obliteration—was observed (7). Yet, Shrestha et al. note that though positive aortic remodelling was evident in patients with thoracic aortic aneurysms, the effect was comparatively less pronounced (7). Similarly, in their recent report on aortic remodelling in 25 patients (8 acute, 13 chronic aortic dissection) treated with Thoraflex Hyrbid™ FET in Birmingham, UK, Mehanna et al. report a promising increase in TL to total aortic diameter ratio during the follow up period, form 0.31 pre-intervention to 0.40 during follow-up (*P* = 0.031). This was accompanied by a decrease in the FL to total aortic diameter ratio during the same period: 0.66–0.54 (*P* = 0.024) (21). No significant difference in aortic remodelling was reported between cases of acute and chronic dissection—suggesting that Thoraflex Hybrid™ is efficacious in inducing positive remodelling regardless of surgical acuity (21).

Interestingly, in their multicentre (in Germany and Austria) comparison of Thoraflex Hybrid™ against E-Vita™ Open, Berger et al. note that patients treated with E-Vita™ Open exhibited more extensive FL thrombosis in the perigraft space relative to Thoraflex Hybrid™ patients (95 vs. 74%, respectively), and that Thoraflex Hybrid™ patients were more likely to require secondary TEVAR (*P* = 0.003) (16). Yet, the authors note that their results are inconsistent with other findings and is likely due to the Thoraflex Hybrid™ grafts used in the study being consistently shorter than the E-Vita™ Open grafts (Thoraflex Hybrid™ and E-Vita™ Open grafts were exclusively used in their 100 and 130 mm configurations, respectively, to attenuate the risk of spinal cord injury) (16). Thoraflex Hybrid™ grafts were also implanted more frequently at Zones 2 and 1 than E-Vita™ Open grafts—proximalising further the distal landing zone (*P* < 0.001). This resulted in shorter coverage of the DTA. Further, at 6 and 12 months post-intervention, FL thrombosis around the coeliac trunk was significantly better in patients treated with Thoraflex Hybrid™ than E-Vita™ Open: 19 vs. 14% and 37 vs. 30%, respectively (16).

A group from Vienna also highlighted that in their series of 27 patients treated for acute thoracic aortic dissection with E-Vita™ Open, FL patency at the level of the diaphragm and coeliac trunk in 52 and 78% of cases, respectively (22). Thoraflex Hybrid™ can therefore be associated with strong rates of positive aortic remodelling, arguably to a greater extent than that other market players. Positive remodelling in patients with Thoraflex Hybrid™ can be augmented by strategic graft sizing, taking proximal anastomosis zone, patient height, and other anatomic factors into account.

In addition to positive and negative remodelling, it is worth considering the risk of in-stent thrombosis associated with Thoraflex Hybrid™. The stent-graft portion of all FET prostheses is designed to seal off any dissection tears within descending aortic intima, however, intraluminal clots causing TL narrowing or downstream malperfusion effect may develop (23). The incidence of in-stent thrombosis is rare irrespective of FET prosthesis used and is sparsely reported in literature. The underlying pathology behind this rare phenomenon has yet to be determined. While reporting their early-to-midterm postoperative results with Frozenix™ deployment at Zone 0 for TAR in patients with ATAAD, Yamamoto et al. highlight that 3 (3%) patients required secondary TEVAR due to TL thrombosis (23). Further, Yoshitake et al. evaluated 426 patients who underwent aortic repair for ATAAD over 11 years. Amongst the patients undergoing FET (*n* = 139), secondary TEVAR was needed in 8 (5.8%) cases due to TL stenosis (24). Finally, Kandola et al. reported only 1 (3%) case of in-stent thrombosis within their population of 36 single-stage FET cases (25). It is crucial to emphasise that there has hitherto been no published evidence suggesting any incidence of in-stent thrombosis following implantation of Thoraflex Hybrid™.

### Neurological Complications

Neurological complications are among the most common and debilitating adverse events associated with aortic arch surgery. This is unsurprising given the necessity for intraoperative circulatory arrest, and the propensity for aortic grafts to occlude branches of the DTA that supply the spinal cord. As a result, cerebral (postoperative stroke, transient ischaemic attack [TIA], cognitive deficit, etc.) and spinal cord manifestations (paraplegia, spinal deficit etc.) may result (9). The pathogenesis of aortic surgery-induced neurological injury is varied and complex; attributing neurological complications to the chosen aortic device, cerebral perfusion technique, device sizing, CPB/HCA duration, or device positioning is therefore challenging.

1.9% (*n* = 18) of patients in the present series suffered postoperative neurological injury. One patient who suffered postoperative paraplegia attributed to spinal cord injury subsequently died on postoperative day 23. This case was attributed to the use of Thoraflex Hybrid™. Seventeen further neurological adverse events were reported across the 84-month follow-up period, including paraplegia, paraparesis, recurrent TIA, cerebral infarct, and recurrent laryngeal nerve (RLN) palsy. None of these subsequent events were attributed to the use of Thoraflex Hybrid™, rather, were described as not device related or procedure related.

Di Bartolomeo et al. Bologna group report similarly promising findings in their experience with Thoraflex Hybrid™ implantation for residual type A dissection, chronic type B dissection, and degenerative aortic arch aneurysm (15). Out of 10 patients treated, zero cases of paraplegia, paraparesis, or major neurological deficit were reported. One patient suffered a postoperative TIA. Similar Thoraflex Hybrid™ case series in Hannover and Munich reported postoperative stroke rates of 10 and 5%, respectively. The Munich group also noted that 13% of patients suffered postoperative phrenic nerve injury (8). Shrestha et al. also noted that 7% (*n* = 7) of patients treated with Thoraflex Hybrid™ (7).

In their Canadian multi-centre analysis of early outcomes following Thoraflex Hybrid™ TAR (*n* = 40), Chu et al. report overall stroke and temporary neurological deficit rates of 5 and 3%, respectively. 5% (*n* = 2) patients suffered transient spinal cord ischaemia, and 15% (*n* = 6) suffered postoperative delirium but had returned to baseline prior to discharge. No cases of permanent paraplegia were reported (4). These findings suggest that Thoraflex Hybrid™ may be associated with significantly improved neurological outcomes relative to market counterparts: Leontyev et al. Leipzig group reported a 19.6% rate of new postoperative paraplegia and a 11.8% postoperative stroke rate following TAR with E-Vita™ Open (26). Our extensive international study over 84 months highlights the long term efficacy of Thoraflex™ Hybrid, with very low rates of neurological complications in comparison.

Though it is challenging to attribute the discrepancy in rates of neurological complications to aortic device alone, it is worth noting that Thoraflex Hybrid's™ unique design—which includes a 4th arch branch to enable early lower body perfusion after distal anastomosis—may help to reduce the risk of spinal cord (and end-organ) ischaemia by reducing the duration of HCA needed (8, 9). Indeed, HCA duration (as well as minimum core temperature and stent graft length) have been identified as predictors of SCI risk, and interestingly, Berger et al. multicentre investigation reported at patients implanted with Thoraflex Hybrid™ underwent substantially shorter HCA durations than those treated with E-Vita™ Open (51 min [41–59], vs. 60 min [51–69], *P* = 0.007) (9, 16). Shrestha et al. highlight that their patients implanted with Thoraflex Hybrid™ underwent HCA for a median of 47 min (36–61) (7).

Extent of DTA coverage by the Thoraflex Hybrid™ stent-graft may also be a determinant of SCI risk (27). Decreased occlusion of the intercostal vessels when shorter stent-grafts are used, or proximalisation of arch repair to Zone 1 or 0 may therefore protect against SCI (28). Yamamoto et al. suggest selective LSA perfusion during HCA as a way to improve intraoperative spinal cord perfusion via collateral vasculature to further mitigate this risk (23).

Further, it is thought that rapid FL thrombosis in patients with chronic aortic dissection potentiates SCI due to the tendency for aortic branches to be supplied by the patent FL. Limiting Thoraflex™ Hybrid stent graft length to 100 mm (assuming a Zone 2 or 3 anastomosis), minimum cooling temperatures of 25°C, and preoperative CSF drainage in elective cases may help to attenuate the risk of spinal cord complications (7).

### Coagulopathy and Reintervention

Coagulopathic complications refer to adverse events involving (or potentiated by) excessive intra- or postoperative bleeding. Frequently, postoperative bleeding necessitates reintervention—a common feature of the postoperative course in aortic surgery. Unfortunately, this further exposes patients to the risks associated with surgery, anaesthesia, and hospitalisation.

In the present series, there were 10 reports of haemodynamic complications within postoperative day 30, 4 of which resulted in death. There were also 2 reports of haemothorax (days 36 and 283 postoperative), and 1 report of persistent type 1b endoleak (identified on day 150 postoperative); though it is unclear what proportion of these patients required reintervention. The Bologna and Hannover groups reported that 20% (*n* = 2) and 13% of their series required reoperation for bleeding, respectively (8). In contrast, the Munich group reported zero bleeding-related complications (8). Shrestha et al. noted that 10% of their cohort underwent rethoracotomy for bleeding (7).

Kreibich et al. report a 33% reintervention rate post-FET; 69% of which involved TEVAR, 20% required open thoracoabdominal aortic aneurysm repair, and 11% required hybrid reintervention (29). Their study identified aortic diameter enlargement, graft endoleak, and dSINE as the most common indications for reoperation—accounting for 44, 23, and 11% of reinterventions, respectively. They emphasise that dSINE formation is particularly dangerous as its onset is typically asymptomatic but potentiates rapid negative remodelling (29). It is likely that prosthesis sizing plays a key role in potentiating endoleak and dSINE formation. The Canadian group reported a 3% (*n* = 1) reintervention rate for bleeding with Thoraflex Hybrid™, and 2 (5%) cases of post-arch repair formation of TBAD—one of which required secondary TEVAR in addition to medical therapy. The authors argue that both instances of postoperative TBAD would have been avoided with less aggressive sizing strategies (4).

It is worth highlighting 2 cases of spontaneous unexpected Thoraflex Hybrid™ stent-graft leakage reported by Kreibich et al., during second-stage thoracoabdominal aortic repair. Upon clamping of distal stented descending aorta, leakage was noted around the proximal untouched stent-graft. Both cases did not exhibit pre-existing stent-graft leaks on imaging (30). In the first case, re-exploration revealed that the stent-graft had become folded while *in situ*. The authors suggest that their unfolding of the Thoraflex Hybrid™ stent-graft lead to disruption of the newly formed neo-intima and neo-adventitia, disrupting tissue incorporation, and causing leakage (30). In the second case, macropores were noted around the stent graft (which eventually required resection and replacement), which the authors suggest may have been an iatrogenic result of aortic cross-clamping during the second stage (30). It should be emphasised therefore that both reports of Thoraflex™ Hybrid prosthesis leakage were very unlikely to be caused by the prosthesis itself, indeed our international study of 931 patients did not demonstrate a tendency toward graft leakage to any effect.

A recent systematic review by Bashir et al. including 6,313 patients treated with Thoraflex Hybrid™, E-Vita™, Frozenix™, and Cronus™ found that publications detailing coagulopathic events following Thoraflex Hybrid™ and Frozenix™ implantation were associated with the least degree of heterogeneity compared to the 3 market alternatives (*I*^2^ = 0.01%, *I*^2^ = 53.95%, *I*^2^ = 0.01%, and *I*^2^ = 54.41%, respectively) (31). Furthermore, while Thoraflex Hybrid™ has hitherto not been associated with major prosthesis-caused coagulopathic events, several recent reports by Ho et al. and Czerny et al. have revealed that E-Vita™ Open NEO has a propensity for catastrophic oozing from the arch graft portion (32, 33). This is likely due to a lack of gelatine impregnation, leaving the graft permeable to blood (11). Pre-implantation priming of the graft with BioGlue (CryoLife, Kennesaw, GA, USA) has been suggested as a strategy to mitigate the risk of graft oozing, however, the suitability and safety of this is highly questionable (11, 32).

### End-Organ Ischaemia

Systemic complications and end-organ damage are omnipresent risks in almost all major surgical procedures, but these are especially pertinent in aortic arch surgery due to the use of HCA and changes to end-organ blood supply following aortic stenting and remodelling. Indeed, this is reflected by the risk of SCI, cerebral ischaemia, and renal injury associated with aortic arch surgery.

Our series includes a total of 37 reports of postoperative complications including multi-organ failure (*n* = 8), cardiorespiratory complications (*n* = 27), renal injury (*n* = 7), and infection (*n* = 13) over the 84-month follow-up period. None of these reports have been associated directly with the use of Thoraflex™ Hybrid; rather, it is likely that these have resulted from procedure-related or hospital-related factors (especially infection). Cardiac complications from aortic arch surgery are varied—it is likely that the use of cardioplegia, circulatory arrest, anaesthesia, and possible iatrogenic occlusion of the coronary sinus may give rise to cardiac events such as myocardial infarct (MI), ventricular dysfunction, pericardial effusion, and new-onset arrhythmias (e.g., atrial fibrillation [AF]). New-onset arrhythmia was reported in 0.1% (*n* = 1) patient included in our series, ventricular dysfunction in 0.6% (*n* = 6), and pericardial effusion in 0.2% (*n* = 2). A further 0.6% (0*n* = 6) patients suffered respiratory failure. 3% (*n* = 1) patient in the Canadian series suffered a postoperative MI, while 25% (*n* = 10) developed new AF, although the patient cohort is significantly limited (4).

Postoperative renal injury—a common complication of many interventions—tends to result from hypoperfusion of the kidneys due to HCA or renal artery occlusion following stenting or aortic remodelling. A total of 0.7% (*n* = 7). patients included in our series suffered postoperative renal injury. Chu et al. found that 3% (*n* = 1) of patients in the Canadian study also suffered renal failure requiring dialysis, while 14% (n = 14) of patients in Shrestha et al. series suffered acute kidney injury (AKI) requiring dialysis (8 patients were discharged on dialysis) (4, 7). Notably, zero instances of renal injury were reported by the Hannover group, while 2 patients in the Munich study required permanent dialysis (8). One patient from the Bologna group suffered renal injury requiring temporary dialysis (8). In contrast, Leontyev et al. highlight that 25.5% (*n* = 13) suffered renal failure post-implantation of E-Vita™ Open, while Song et al's. report zero instances of AKI following implantation with Cronus™ (18, 26).

Because downstream pathology is not uncommon in patients undergoing TAR with FET, is it crucial that surgeons assess the presence of distal re-entry tears downstream. Surgical planning around the presence of distal re-entry tears may minimise the risk of FL thrombosis-induced end-organ perfusion, especially in cases of chronic TBAD (7). Interestingly, the development of custom-made branched or fenestrated endovascular aneurysm repair (EVAR) grafts may pave the way toward mitigating visceral perfusion in endovascular and open aortic repair.

## Conclusion

Having appraised the design, reported outcomes, and published literature concerning the international clinical efficacy of Thoraflex Hybrid™ as an FET graft, it is clear that the device is associated with excellent usability, favourable mortality and aortic remodelling rates, as well as relatively low rates of postoperative complications. The risk-benefit profile of Thoraflex Hybrid™ is all the more favourable when viewed in the context of available market alternatives and off-brand FET techniques.

## Data Availability Statement

The original contributions presented in the study are included in the article/supplementary material, further inquiries can be directed to the corresponding author/s.

## Ethics Statement

Ethical review and approval was not required for this study with human participants, in accordance with the local legislation and institutional requirements.

## Author Contributions

ST, MJ, and MB were involved in the drafting of the manuscript. MB and IM were involved in reviewing and providing feedback on the manuscript. All authors contributed to the article and approved the submitted version.

## Conflict of Interest

The authors declare that the research was conducted in the absence of any commercial or financial relationships that could be construed as a potential conflict of interest.

## Publisher's Note

All claims expressed in this article are solely those of the authors and do not necessarily represent those of their affiliated organizations, or those of the publisher, the editors and the reviewers. Any product that may be evaluated in this article, or claim that may be made by its manufacturer, is not guaranteed or endorsed by the publisher.
